# The everyday speech environments of preschoolers with and without cochlear implants

**DOI:** 10.1017/S0305000924000023

**Published:** 2024-02-16

**Authors:** Margaret CYCHOSZ, Jan R. EDWARDS, Benjamin MUNSON, Rachel ROMEO, Jessica KOSIE, Rochelle S. NEWMAN

**Affiliations:** 1 University of California, Los Angeles, USA; 2 University of Maryland, College Park, USA; 3 University of Minnesota, Twin Cities, USA; 4 Princeton University, USA

**Keywords:** cochlear implant, deafness, language input, spoken language, language interaction

## Abstract

Children who receive cochlear implants develop spoken language on a protracted timescale. The home environment facilitates speech-language development, yet it is relatively unknown how the environment differs between children with cochlear implants and typical hearing. We matched eighteen preschoolers with implants (31–65 months) to two groups of children with typical hearing: by chronological age and hearing age. Each child completed a long-form, naturalistic audio recording of their home environment (appx. 16 hours/child; >730 hours of observation) to measure adult speech input, child vocal productivity, and caregiver-child interaction. Results showed that children with cochlear implants and typical hearing were exposed to and engaged in similar amounts of spoken language with caregivers. However, the home environment did not reflect developmental stages as closely for children with implants, or predict their speech outcomes as strongly. Home-based speech-language interventions should focus on the unique input-outcome relationships for this group of children with hearing loss.

## Introduction

Children learn the sounds and structure of their native language(s) from the input that they receive from caregivers around them. Yet children vary in the type and quantity of their speech-language exposure, including the number and diversity of word types (Rowe, 2012), ratio of male to female speakers (Bergelson et al., 2019), amount of child-directed speech (Rowe, 2008; Schwab & Lew-Williams, 2016), and acoustics of caregiver speech (Dilley et al., 2014). Crucially, these individual differences in linguistic exposure may account for some differences in children’s speech-language development. For example, 6 to 14-month-olds who engage in more contingent interactions with caregivers – typically defined as conversational exchanges that take place in quick succession (≤ 2 seconds) – produce more babbling sounds and grow larger expressive and receptive vocabularies (Ferjan Ramírez et al., 2020). Similarly, infants who hear more child-directed speech at 7 (Newman et al., 2016) or 19 months (Weisleder & Fernald, 2013) learn to process words more efficiently, helping them grow larger vocabularies by age two.

This causal link between individual differences in linguistic input and speech-language development, which persists independent of family socioeconomic status or caregiver characteristics (Romeo et al., 2018), likewise extends to non-typically developing groups such as children who receive autism spectrum disorder diagnoses (Swanson et al., 2019) and, most critically for the current work, children with hearing loss, including those who receive cochlear implants (CIs) (Ambrose et al., 2014, 2015; Arjmandi et al., 2021; DesJardin & Eisenberg, 2007; Dilley et al., 2020; Wang et al., 2020). For example, Dilley et al. (2020) correlated acoustic-lexical properties of speech directed to a cohort of recently-implanted infants (8–29 months at implantation) and found that more diverse word types, and more dispersed vowel spaces in infant-directed compared to adult-directed speech, predicted higher scores on standardized language assessments two years post-implantation (see also Wang et al., 2020). Elsewhere, caregivers of children with CIs who had greater mean lengths of utterance, and used more open-ended and/or recast questions, likewise had children with higher standardized receptive and expressive language scores (DesJardin & Eisenberg, 2007). Although standardized assessments never capture the full complexity of a child’s linguistic development, these results do show that individual differences in linguistic input to children with CIs can predict some of the well-acknowledged variation in implantees’ developmental outcomes. As such, having a clear characterization of these children’s everyday speech-language environments is fundamental to understanding their speech and language development.

### How cochlear implantation might shape the early language environment

There are two components of cochlear implantation that may systematically alter child implantees’ speech-language environments (Houston, 2022). In the United States, the FDA has approved cochlear implantation for infants as young as 9 months (USFDA, 2020) (and some children may be implanted off-label even earlier). However, some children may not receive one or both of their CIs until their second or third birthdays (Warner-Czyz et al., 2022). Regardless, even assuming the earliest implantation age, children experience an extended period of auditory absence pre-implantation where they are not exposed to spoken language models and caregivers may direct less spoken language to them. Second, once CIs are activated, recipients have the added challenge of compensating for signal degradation: CI electrode arrays stimulate the cochlea at discrete points, discretizing the speech envelope. There are additional issues inherent to the hardware such as electrode interaction and interaural mismatch, as well as physiological aspects such as irregular neuronal survival, that together degrade the auditory signal presented to the child. Children vary greatly in how they are able to adapt to this degraded signal to learn speech and language. CIs also only introduce the sensation of hearing when the devices are being worn, and children vary greatly in the number of hours of typical device use (Ganek et al., 2020).

#### How auditory absence shapes the early environment

Pediatric CI candidates must pass a series of candidacy requirements (e.g., >70dB pure-tone thresholds in both ears, limited benefit from acoustic amplification, typical anatomical cochlear development). Although the FDA permits implantation in children as young as 9 months, recipients are often only implanted between 24 and 36 months (Warner-Czyz et al., 2022). Prior to implantation, spoken language is often used in the home (even if many aspects of it are inaccessible to the child), especially if the child is born to hearing parents. Additionally, some children are exposed to one or more forms of signed languages.

The absence of auditory input pre-implantation can impact the speech-language environments of children with severe to profound hearing loss. In infancy, children with typical hearing (TH) who vocalize more receive more contingent responses from caregivers (and vice versa) resulting in a social feedback loop that spurs early speech development (Warlaumont et al., 2014). Yet despite a common saying that “*even children who are deaf babble*” (orally), prior to intervention, children who go on to receive CIs vocalize infrequently and babble immaturely (Fagan, 2014). (Early evidence for mature babbling among children with hearing loss is often confounded with the degree of hearing loss.) In reality, it takes many months *post*-implantation for pediatric CI recipients to produce speech on par with their peers with TH and even longer to engage in socially-contingent linguistic interactions with caregivers (Fagan et al., 2014; Kondaurova et al., 2020), demonstrating how auditory absence may shape the early linguistic environment.

#### How signal degradation shapes the early environment

The signal transmitted by the CI is degraded in ways that may clearly implicate and shape children’s speech-language environments. CI users of all ages have reduced access to temporal fine structure cues, such as those that encode the fundamental frequency (f0) of the voice (the perceived pitch of the voice) and reflect speaker pitch or lexical tone (Chatterjee & Peng, 2008; Lee et al., 2002). Yet children with TH use pitch cues to help differentiate between speakers in their environment (Nagels et al., 2021). Since children with CIs do not have the same access to these cues, certain aspects of speech processing such as normalization for speaking rate or vocal tract length, as well the ability to segregate overlapping speakers, prove more difficult for them (Cleary & Pisoni, 2002; Nittrouer et al.,, 2014). In addition, interaural differences in CI electrode insertion depths result in different frequency-to-place mismatch interaurally, compromising sound localization cues (see evidence in middle childhood: Todd et al., 2016). (Reduced sound localization cues are also due to lack of experience with acoustic hearing (for pre-lingually deafened children) and lack of experience with binaural hearing (Grieco-Calub & Litovsky, 2012) (e.g., for single-sided deafness).) Deficits in localization cues can make it more difficult for children to LOCATE the source of a caregiver’s voice – and a failure to look towards a speaker could alter the feedback loop that drives caregiver speech input (Wang et al., 2017). The deficits may also make it difficult for children to ATTUNE to audio-visual cues from the lips that may help compensate for a degraded auditory signal (Bergeson et al., 2005).

Altogether, the degraded CI signal, including compromised f0 cues and interaural frequency-to-place mismatch, may shape children’s daily language environments and interactions with caregivers: the degraded signal affects how children attune to linguistic input, potentially explaining some of the differences between children with CIs and TH in child-caregiver vocal contingency, and synchronization during joint attention (Chen et al., 2019), even a year post-implantation.

### Current study

The purpose of this study was to evaluate how the listening experiences of children with CIs shape their everyday speech-language environments. To evaluate this, we matched a cohort of 3- to 5-year-olds with CIs separately to a group of their hearing age- and chronological age-matched peers. We densely sampled the children’s naturalistic home environments using child-friendly wearable recording devices (appx. 16 hrs./child or >730 total hours of observation) allowing us to assess a battery of characteristics of the home speech-language environment. Specifically, we characterized the quantity, consistency, and experience-related differences in children’s speech input, vocal output, and conversational interactions with caregivers. Finally, we assessed how the children’s speech-language input predicted their vocal productivity. Below we outline our predictions for how each metric may differ for children with CIs and TH:

1. *Why might quantity of caregiver input differ?* Children with CIs could be exposed to more caregiver input than hearing age (HA) matches because they are at a more advanced cognitive developmental stage, and caregivers of children with TH use more word tokens and complex grammatical structures as children develop (Huttenlocher et al., 2007). Or, alternatively, caregivers could talk more to children with CIs in an attempt to compensate, believing the child needs more exposure. Children with CIs may receive less caregiver input than chronological matches, however, if caregivers are sensitive to the children’s developing linguistic capabilities, especially in the first months and years post-implantation (DesJardin & Eisenberg, 2007).
2. *Why might child vocal output differ?* The vocal output of children with CIs is likely to be less mature than chronological age matches (less frequent, shorter duration) because the children with CIs have had less experience incorporating auditory and somatosensory feedback from their own speech production due to the time spent without access to sound pre-implantation (Fagan, 2014). Signal degradation from the CIs post-implantation may also make it harder for children with CIs to establish reliable speech-motor maps, thereby delaying progression through later stages of speech development, and resulting in less mature or error-prone vocal production several months post-implantation (Serry & Blamey, 1999). (Children’s vocalizations are also part of their auditory input so less mature vocalizations mean that the child would also be receiving input that is less mature.) Yet, children with CIs would be expected to have more mature vocal output than HA matches because their speech-motor apparatuses are more mature and capable of sustaining phonation, and they are also more able to coordinate inter-articulatory movement such as the tongue and the jaw (Smith & Goffman, 1998).
3. *Why might conversational interactions differ?* The children with CIs had less opportunity to establish vocal contingency patterns (although not necessarily other forms of contingency such as gaze – Chen et al., 2020) pre-implantation, in infancy (Northrup & Iverson, 2020). Among children with TH, these early contingency behaviors are highly correlated with later linguistic outcomes (Donnelly & Kidd, 2021; Hirsh-Pasek et al., 2015), so the reduced interactions in infancy could set the stage for different quantities or types of conversational interactions even post-implantation. Such a result would mean that at least early after gaining access to sound, the children might pattern like the HA matches, not chronological.

One might assume that chronologically older children would engage in more conversational interactions, because they have the cognitive and linguistic skills to support the exchanges, in which case children with CIs would engage in more conversational turns than HA matches. However, children with CIs have actually been found to engage in *fewer* turns than HA matches, perhaps because their older age grants them more bodily autonomy, distance from caregivers, and opportunity to interact with other interlocutors such as siblings. This is the result concluded by Kondaurova et al. (2022), and indeed conversational turn development among children with TH follows this developmental pattern – initially increasing in toddlerhood but decreasing in the preschool years – through 48 months of age (Gilkerson et al., 2017). Signal degradation of, for example, f0 cues, may also play a role. Children with CIs may be less attuned to cues for speaker location, interlocutor identity, and utterance polarity (questions are produced with rising f0 contours and statements with flat or downward sloping contours and/or a creaky voice modality), all causing children with CIs to respond or engage less readily with caregivers in their environments.

- *Why might the relationship between speech-language input and child vocal productivity differ?* Children with TH who hear more speech and/or engage in more conversational turns have more mature speech production outcomes in infancy and early childhood (Ferjan Ramírez et al., 2020; Ruan, 2022). However, this relationship may be less predictive for children with CIs than children with TH. First, factors such as device and implant properties, as well as auditory training, may play outsize roles for the vocal maturity of children with CIs, rendering caregiver input less predictive of the children’s outcomes. Second, children with CIs could have more difficulty locating caregivers in the environment due to signal degradation and device limitations (Houston, 2022), making it more difficult for them to separate speech from other sounds and process words (Vavatzanidis et al., 2018), and potentially making conversational interactions less predictive. Finally, signal degradation from the devices could lower the quality of speech input that children with CIs receive, so hearing more input may not be as beneficial for CI users as children with TH.

## Materials and Methods

### Participants

Fifty-two children participated in this study. The N = 18 children with CIs were individually matched by parent-reported gender, socioeconomic status, and age to two groups of children with TH: (1) chronological age matches, to match for cognitive and articulatory development (N = 18), and (2) hearing age matches, to match for auditory experience (N = 16 as 2 children with CIs had <1 year of hearing experience). Hearing age was computed as the difference between the child’s current, chronological age and the date of their first implant activation. All children were monolingual English speakers and age matching was made within 3 months whenever possible. Table 1 presents demographic information by hearing group. There were no reliable differences in number of siblings (F(2) = 1.16, p >. 05) or household members (F(2) = 0.98, p > 0.05) by hearing group. See Supplementary Materials I for detailed reports on family composition by hearing group. This work was approved by the relevant institutions’ Institutional Review Boards.

Socioeconomic status was instantiated as the highest level of maternal education achieved. To facilitate matching, we binned education into seven levels: 1) < high school, 2) high school equivalent certificate (e.g., General Education Development [GED]), 3) high school diploma, 4) technical-associate degree, 5) some college (2+ years)/ trade school, 6) college degree, and 7) graduate degree. All maternal education matching was made within 1 degree of freedom (i.e., caregiver with level 3 education preferentially matched to caregiver with level 3, and if not then matched to level 2 or 4).

N = 14 children used bilateral CIs, N = 2 unilateral, and N = 2 had a bimodal CI +hearing aid configuration. The children with CIs had hearing parents and were being schooled in auditory-verbal (N = 11), auditory-verbal+aural focus (N = 2), aural focus (N = 1), cued speech (N = 1), or auditory-verbal+total communication (N = 1) environments (auditory-verbal programs can also be referred to as listening and spoken language). Data on school environments were unavailable for two children. See Appendix A for detailed, by-child audiological information. All children with TH had parent-reported typical speech-language development.

### Data collection

Each child completed one daylong audio recording where they wore a small, lightweight Language ENvironment Analysis (LENA) recording device (2”x3”; 2 oz.) in a specialized vest for an entire day. Recordings were completed on a typical, non-school day in the child’s life. Families were instructed to turn the device on in the morning when the child awoke and continue recording for the duration of the device battery (16 hrs.). During bathtime and other water activities, parents were told to place the recorder in a safe, dry place as close to the child as possible. All families except one completed the full 16-hour recording; the remaining family completed a 12.83-hour recording. In all cases, the device continued recording while the child napped.

### Audio processing

Measures of the children’s home speech-language environments were semi-automatically derived from each child’s recording using LENA’s diarization algorithm which assigns speaker tags and timestamps to audio clips (Xu et al., 2009). Speech clips tagged as “Target child” (CHN), “Male adult near” (MAN), and “Female adult near” (FAN) were extracted. We filtered out CHN clips that contained cries, FAN/MAN clips that contained any non-speech, and FAN/MAN clips > 10s (574 clips, 0.07%). In our experience these clips longer than 10s tend to be mislabeled. The decision to remove all FAN/MAN clips that contained *any* non-speech elements inevitably resulted in the removal of some adult speech near the child, but the step was maximally conservative and allowed us to be sure that the adult speech clips in the final analysis contained only speech. All audio processing scripts are included in the project’s Github repository (https://github.com/megseekosh/everyday_CI).

**Figure 1.**
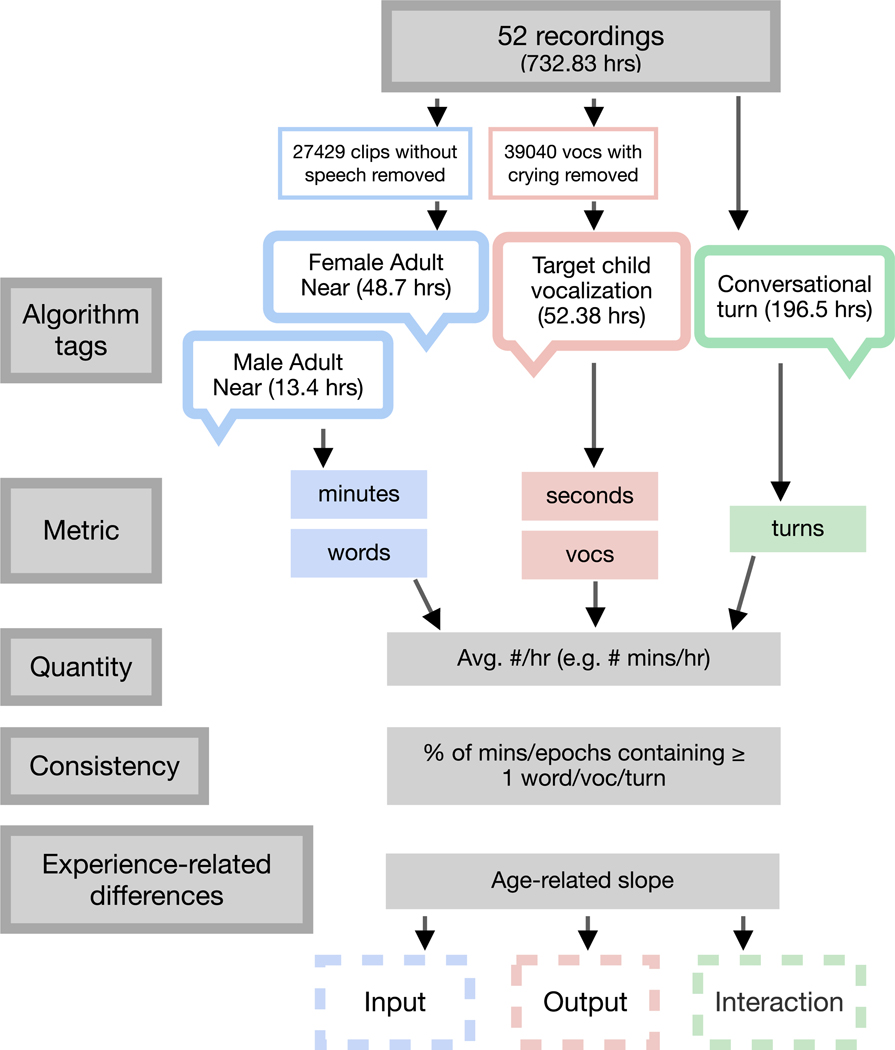
Daylong audio recording processing steps for the primary analysis. Hours reflect totals after removing clips without speech/crying.

Word token count estimates from the adult clips were likewise extracted. For simplicity, in the remainder of the manuscript, we will refer to the FAN and MAN clips as “caregiver speech,” although we stress that the clips could have contained speech from a non-caregiver adult who was speaking within 10 feet of the child. Finally, algorithmic estimates of “conversational turns” were extracted. These were defined as target child and adult utterances spoken within 5 seconds, not conversations between e.g., two adults or an adult and another child. See Figure 1).

Speech input was modeled in minutes (total duration of FAN+MAN clips) and number of words from adults. Speech output was modeled in seconds (duration of CHN clips) and the number of vocalizations from the child. Seconds were used, instead of minutes as in the adult speech, because the child vocalizations were typically much shorter than the adults’ speech. Caregiver-child interaction was instantiated as the number of conversational turns. We normalized all measures by hour to account for time-of-day differences, as well as different recording lengths. To derive *quantity* estimates of each construct, we computed the average number of (words, vocalizations, turns) per hour. For consistency estimates, we followed King et al. (2021) in computing the percentage of units containing at least one measure (speech input: percentage of minutes in the recording containing ≥ one adult word; speech output: percentage of minutes containing ≥ one child vocalization). For interaction, we computed the percentage of 5-minute epochs containing at least one conversational turn. Finally, to derive experience-related differences in the estimates, we computed the slope of the relationship between child age (in months, either chronological or hearing) and each measure. We elaborate upon this modeling in the results. Together, this workflow allowed us to assess the quantity, consistency, and experience-related differences of various metrics of the children’s speech-language environments.

There has been substantial work evaluating the LENA system’s algorithmic performance for children with and without hearing loss learning English (Cristia et al., 2020; Gilkerson et al., 2017; Lehet et al., 2021; VanDam & Silbert, 2016). (It is beyond the scope of this paper to report on validity and reliability of all LENA metrics so we refer readers to those citations.) Crucially, our analyses relied on diarization and tags that have relatively high recall and precision for the language and age group studied (e.g., “Female adult near” > 60% for English-learning infants and preschoolers (Cristia et al., 2020)) and not those categories, such as electronic speech, that have poorer reliability. Nevertheless, as algorithmic performance is not exact for any of the analyzed categories, we interpret our results by comparing *across* hearing groups. There should be no reason why algorithmic performance would be better or worse by hearing group, and we stress that reports of exact amounts of e.g., words, vocalizations, or turns per hour, should be interpreted with caution (Ferjan Ramírez et al., 2021; Lehet et al., 2021).

## Results

We divide the results section into the various components of each child’s daily speech-language experience: (1) caregiver input, (2) target child output (production), and (3) caregiver-child conversational turns – and evaluate the impact of hearing group upon each outcome. See Table 2 for summary statistics of the measures.

Data were analyzed in the RStudio computing environment (R version 4.2.1; RStudioTeam, 2020). All computing and statistical analyses are included in the GitHub repository affiliated with this project (https://github.com/megseekosh/everyday_CI). Visualizations were made using ggplot2 (Wickham, 2016) and modeling was conducted using lme4 and lmerTest packages (Bates et al., 2015; Kuznetsova et al., 2017); see project documentation for package versions. All model fitting began with a baseline, random-effects only model. Model fit improvements were evaluated by comparing model log-likelihood values and AIC estimates. Unless noted otherwise, the predictor Hearing Group (3-levels: CI, chronological age matches, hearing age matches) was contrast-coded with ‘CI’ as the reference level so model coefficients for the chronological and hearing age match groups refer to deviance from the CI group as this is our main comparison of interest. All continuous variables were mean-centered for modeling but visualizations present the unscaled data. For all examples of repeated measures, such as child vocalization duration (i.e., a duration measure was taken from each child vocalization), we fit linear mixed effects models with random intercepts by child and a fixed effect of **Hearing Group.** Models of hourly measures (words, minutes) additionally included random intercepts by hour of recording.

### Input

We quantified the children’s speech-language input by computing the average number of minutes/hour that contained speech from an adult female or male near the child. We additionally computed the average number of words/hour spoken by an adult near the child.

**Figure 2.**
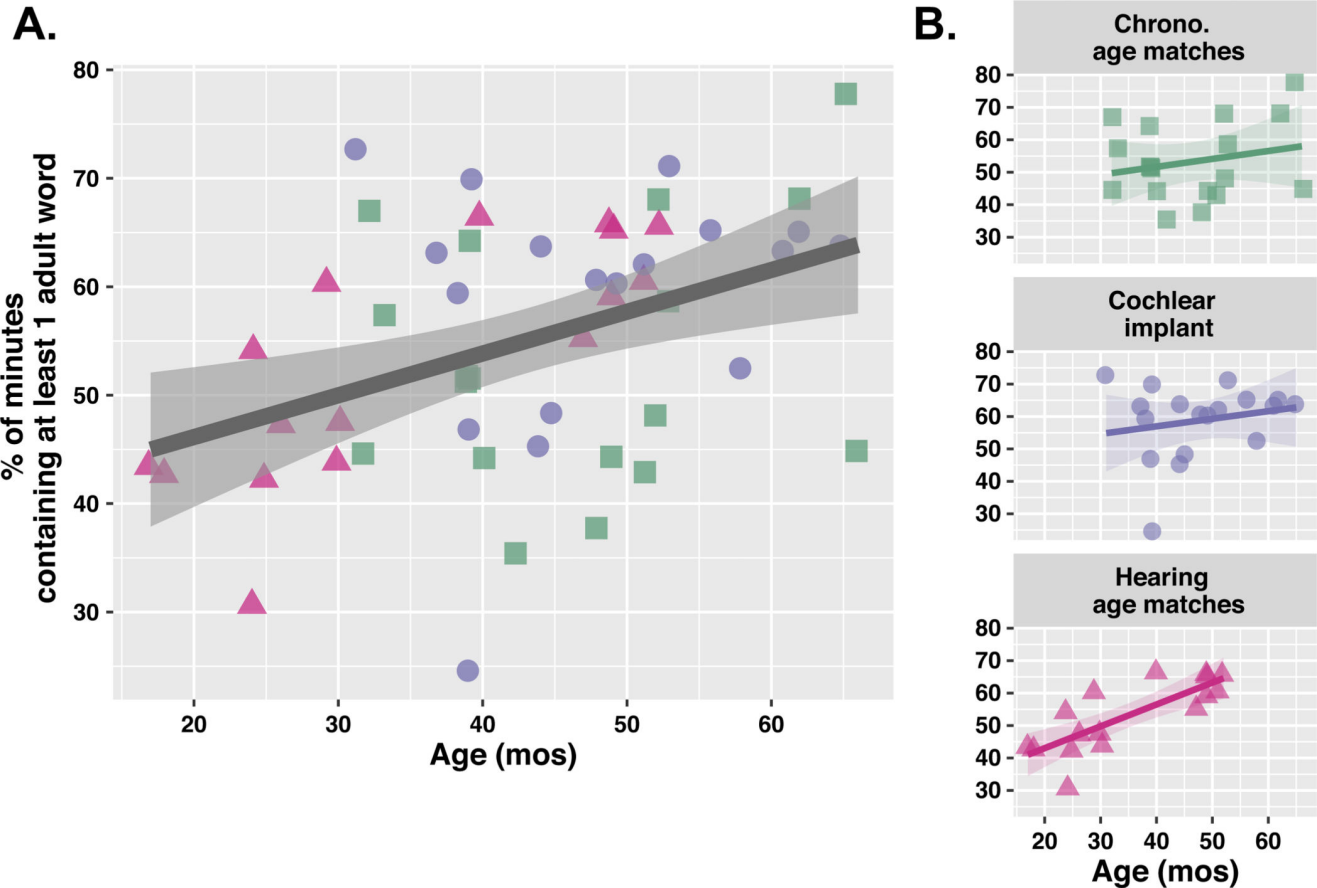
Cross-sectional analysis of speech input consistency across entire sample (A) and by hearing status (B). Each point represents one child. Dark, gray regression line represents local regression fit to all children; ribbons represent 95% confidence intervals. Speech input becomes more consistent, and less bursty, in older children with no interaction by hearing status.

There were no reliable differences by **Hearing Group** for measures of input quantity (hourly words, hourly minutes of speech; log-likelihood tests all *p* >.05); thus, all groups received similar amounts of input in the environment (words and minutes). We evaluated the consistency of speech input by hearing group by computing the percentage of minutes in each recording containing ≥ 1 word from an adult (King et al., 2021). There were no differences in speech input consistency by **Hearing Group** (*p* >.05). However, speech input became more consistent with **Child Age** (age coded continuously, in months) across the entire sample, independent of hearing status (model fit: *β* = 0.004, t = 3.16, *p* = .003). This result indicates that speech is more continuously present throughout the day in older children (Figure 2). Note that this measure of consistency is independent of speech quantity, or the overall *amount* of speech input (words or minutes). Speech input is more consistent – more evenly spread out and less clustered into bursts over the course of the day – in older children across the sample.

Finally, we evaluated differences by hearing group in a cross-sectional analysis of speech input by age. For this analysis, we modeled the effect of **Child Age** (in mos) upon hourly adult word token count and minutes of adult speech/hour in the children’s environments.

Naturally, children with CIs vary along three factors: chronological age, hearing age/experience, and activation age/duration of deafness. These three measures are so highly interrelated in childhood (i.e., a child with an older activation age has less hearing experience) that here we model only by chronological and hearing age (time since activation). Effects of hearing age were only modeled for children with ≥ 12 mos of hearing experience (see Methods). Additionally, there was one clear outlier in age of activation (activated at 45 mos; this child also only had 8 mos hearing experience); in supplementary materials III, we replicate all modeling results with this child removed to ensure that our effects were robust to the outlier.

Hourly word counts and minutes of speech/hour increased with child age in both groups of children with TH, but not by hearing or chronological age among the children with CIs (Table 3): for every month of development, chronological age matches (spanning 32–66 mos) received approximately 21 additional words/hour and 5 additional seconds of speech/hour while hearing age matches (17–52 mos) received an additional 16 words/hour and 4 seconds of speech/hour. Again, no such cross-sectional effect by age was seen for the children with CIs, meaning that unlike children with TH, the quantity of speech input does not reflect child age (hearing or chronological) as well among children with CIs.

### Output

To assess each child’s speech output (production), we computed the average number of vocalizations from the target child spoken/hour. We additionally analyzed the impact of hearing group upon the duration of children’s vocalizations. For the repeated measures (vocalization duration), we fit linear mixed effects models with random intercepts by child and a fixed effect of **Hearing Group.** Models of the hourly vocalizations additionally included random intercepts by hour of recording. There was no effect of **Hearing Group** on the number of vocalizations/hour (*p* >.05); so, hearing status did not dictate the amount of the children’s speech. However, there was an effect of hearing status in the model predicting vocalization duration (comparison of models with and without **Hearing Group**: *χ*^2^ = 6.95, df = 2, *p* = .03): the chronological age matches produced significantly longer vocalizations than both the children with CIs (*β* = 59.25) and hearing age matches (*β* = 78.71).

We additionally measured the consistency of children’s speech output which we quantified as the percentage of minutes in each recording containing at least one vocalization from the target child; there was no effect of hearing experience upon children’s vocalization output consistency.

**Figure 3.**
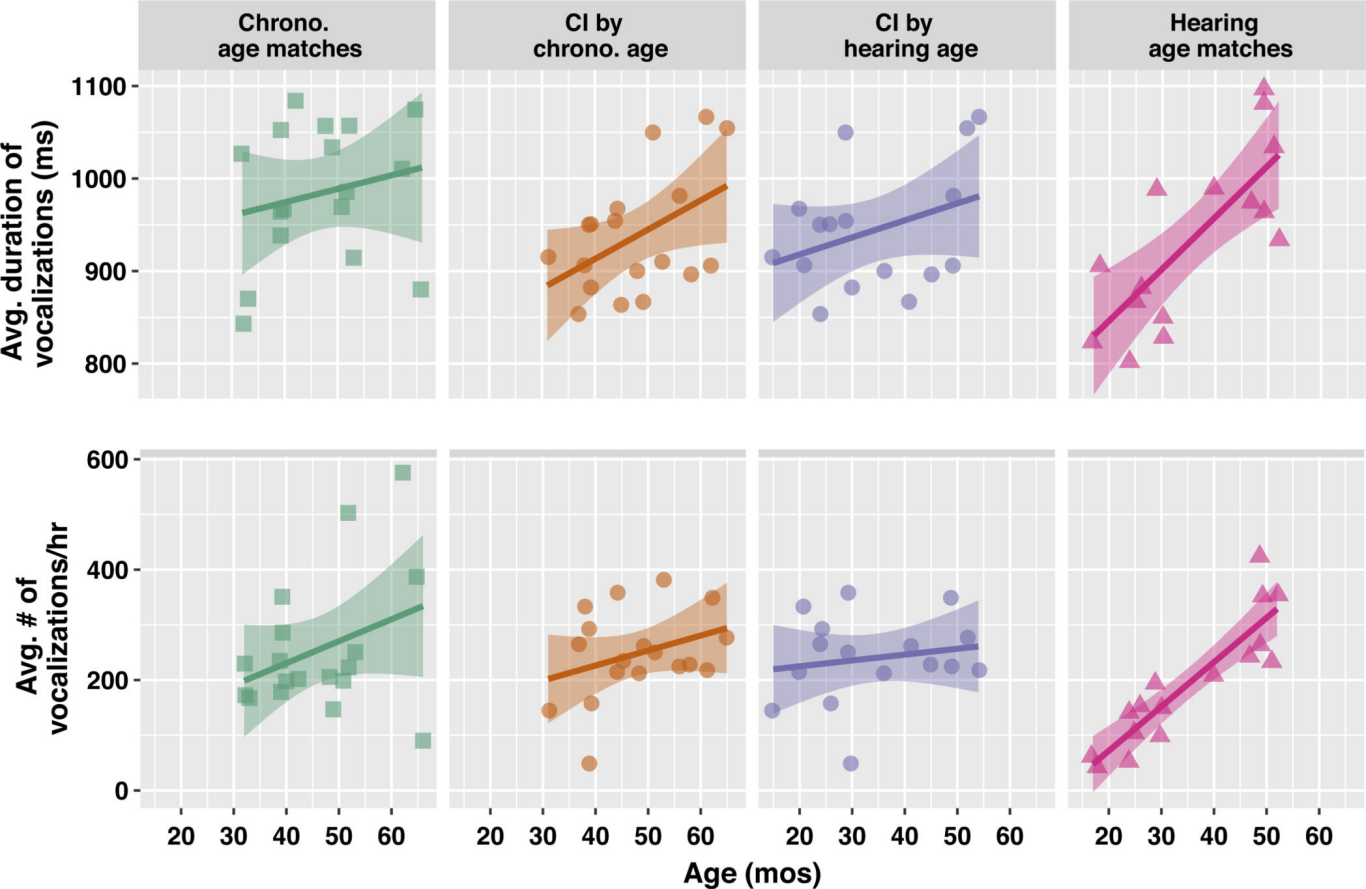
Cross-sectional analysis by age in child vocalization duration (top) and vocalization quantity (bottom), by hearing group. Given the number of distinct data points (N=167130 across all children), local regression lines are fit to averages over each child; ribbons represent 95% confidence intervals. See Table 3 for exact model fit statistics for each outcome.

Finally, we measured the cross-sectional differences by age in vocalization quantity and duration: there was a significant, positive effect of **Child Age** (mos) on vocalization duration among the children with CIs by chronological age, and for the hearing age matches (Figure 3). With each additional month, the duration of the children with CIs’ vocalizations increased by approximately 3.16ms, a shallower slope than for the hearing age-matched children with TH (6.59ms/month; Table 3).

#### Caregiver-child interactions

We next evaluated the impact of hearing group upon caregiver-target child conversational turns. There was no effect of **Hearing Group** upon the quantity or consistency of turns (both *p* >. 05). The cross-sectional analysis by age showed a positive relationship between age and conversational turn quantity only for the hearing age matches (e.g., for the youngest children).

#### Predicting vocal productivity from input measures

**Figure 4.**
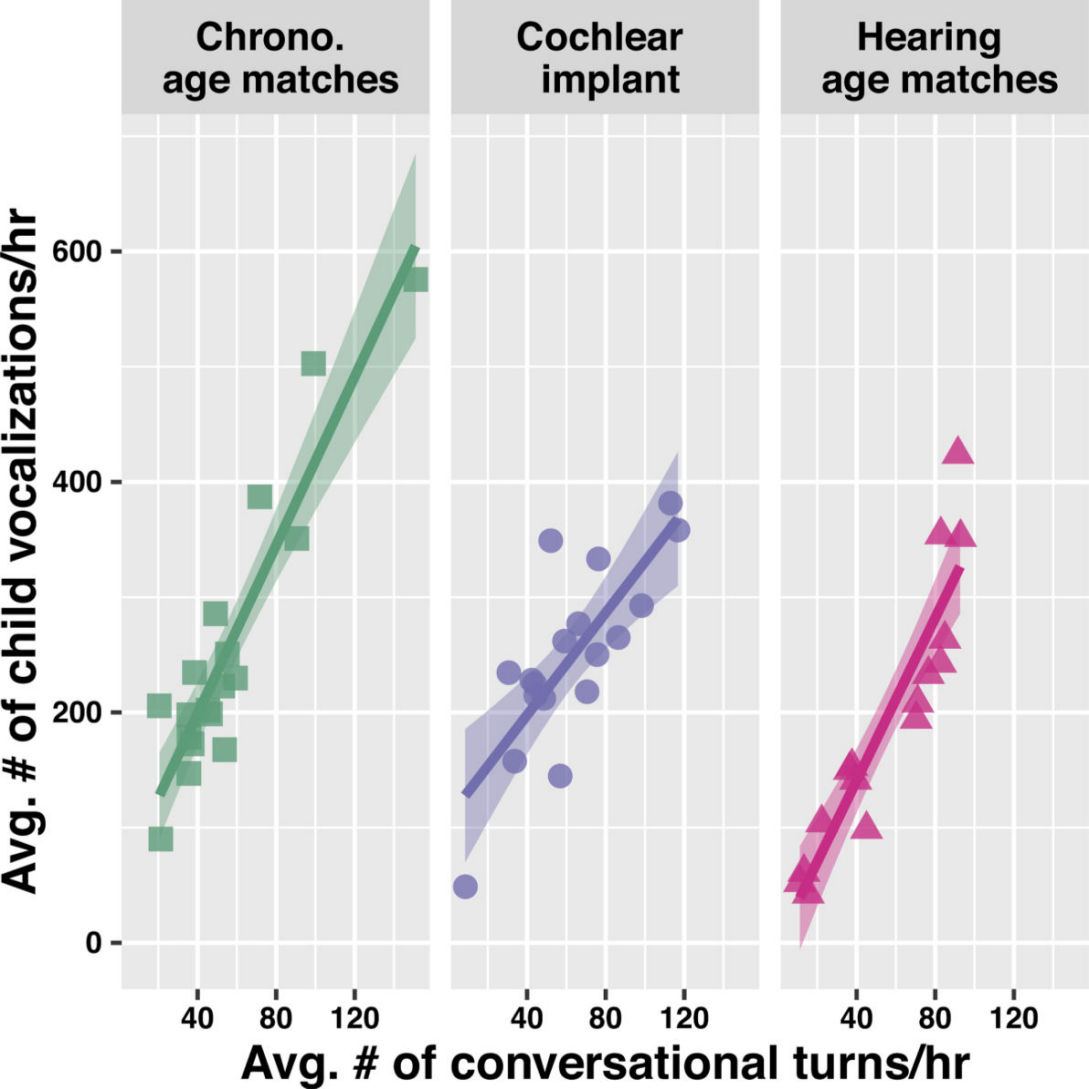
Relationships between child vocal productivity and conversational turns between adult and child. Each point represents the averaged values from one child’s recording and dark lines represent the local regression around those values; ribbons represent 95% confidence intervals. The relationship between hourly conversational turns and child vocal productivity is weakest for the children with CIs.

For the final analysis, we examined how the speech environment predicted children’s overall speech productivity and how this relationship varied by hearing group. It is expected that children who hear more speech, and engage in more linguistic interactions with caregivers, should vocalize more (Ferjan Ramírez et al., 2020; Ruan, 2022; Warlaumont et al., 2014), but it is unclear how the strength of this relationship varies by hearing status and experience. For this analysis, the measure of input that we examined was the **Average number of conversational turns/hr** and the measure of speech productivity used was the average number of target child vocalizations/hour in each recording (Figure 4).

We fit a linear regression model, controlling for chronological age (in mos), to predict the average number of target child vocalizations per hour in each recording from the parameter **Average number of conversational turns/hour.** Then, we evaluated how the relationships between input and child vocal productivity might differ by hearing status. The interaction of **Average number of conversational turns/hour** and **Hearing Group** improved upon a model without the interaction (model fit comparison: *χ*^2^ = 3.72, df = 2, *p* = .03), suggesting differences in the predictive strength of conversational turns for children with CIs. Specifically, for every additional conversational turn per hour that children with CIs engaged in, they produced approximately two additional vocalizations per hour (*β* = 2.22, t = 5.28, *p*<.001). However, this relationship between turns and child vocal productivity was significantly steeper for both groups of children with TH who produced approximately 3 or 4 additional vocalizations per hour for every hourly conversational turn that they engaged in (chronological matches: *β* = 1.44, t = 2.53, *p* = .02, or a slope of 3.66; hearing age matches: *β* = 1.31, t = 2.18, *p* = .03, or a slope of 3.53), suggesting differences in the predictive nature of language in the home environment for children with CIs. At this point, we want to emphasize the relatively large chronological age range of our CI participants (31–65 months) and stress that in a cross-sectional design it can be difficult to reliably evaluate the contribution of age/development (as compared to individual variation) upon these outcome measures. Only longitudinal designs can reliably track the course of development. We discuss ways to responsibly interpret these limited data further in the Discussion.

## Discussion

Results from this study can be distilled into two main findings. First, *the language environment does not appear to reflect development as closely for children with CIs.* Unlike children with TH, older children with CIs do not hear more speech than younger children with CIs (in hearing or chronological years). Hourly conversational turns and turns were less predictive of vocal productivity for children with CIs than either group of children with TH.

Second, *children with CIs engage in just as much caregiver-child vocal interaction as children with TH.* Decades ago, much research suggested that children with hearing loss interacted less with caregivers (Lederberg & Mobley, 1990; Meadow-Orlans & Steinberg, 1993), something that we found no evidence for in our analyses of children with CIs here. However, it is important to acknowledge just how much has changed for children with hearing loss since those seminal studies: the last 30 years have seen the rise in universal newborn hearing screenings, for example, and cochlear implantation is accessible to children within the first year of life. Consequently, while some recent evidence from more controlled, lab-based observations of parent-child interaction still suggests that children with CIs vocally engage less with caregivers (Kondaurova et al., 2020), it is possible that the changes in access to hearing intervention and technology have closed the gap in quantity of parent-child interaction between children with and without hearing loss. And these changes may be especially notable in naturalistic observations of parent-child interaction. In the following sections, we explore both of these points in detail and situate the results in the context of previous work, especially work involving lab-based samples.

### Caregiver speech input

The amount of caregiver speech in the children’s environments was predicted to differ by hearing status. Perhaps the children with CIs would hear more speech input than the hearing age matches (due to differences in cognitive maturity) but less than the chronological age matches (due to differences in linguistic ability). The final picture was more complex: although all groups received similar AMOUNTS of adult speech input, this differed systematically by age. In typical development, children hear more word types, tokens, and overall amounts of speech as they progress from infancy to preschoolhood (Glas et al., 2018; Rowe, 2008) – we replicated these differences by age in cross-sectional samples in both of our TH groups. However, there were no such cross-sectional age differences for the children with CIs, not by hearing age or chronological age. Again, it is not the case that children with CIs simply hear more speech in general and are thus “saturated,” with little room for differences by age-there were no differences in overall input quantity by hearing group. Instead, we take this as the first piece of evidence that the language environments of children with CIs may reflect the individual child less (in this case the child’s hearing or chronological age) than the environments of children with TH. Alternatively, the linguistic and cognitive development of children with CIs may correlate less strongly with age, chronological or hearing, and more with another developmental index unique to this population, such as the combination of hearing age and CI device performance or hours of daily use. In that case, caregivers of children with CIs may attune their input to, for example, the child’s CI experience, which would explain the lack of an age effect in the analyses. Finally, the CI sample in this study had a relatively wide chronological age range. This range can make it difficult to assess how metrics such as number of adult words or seconds of input change over development. And cross-sectional designs, by their nature, cannot track development over time, they can only infer it. Thus, while these results suggest differences in the relationship between age and the outcome variables by hearing group, we encourage future research on this topic in large, more dense samples to ensure the robustness of this conclusion.

### Child vocal output

As predicted, the children with CIs produced shorter vocalizations than chronological age matches; crucially, however, they did not vocalize less OVERALL than either TH group and had some cross-sectional differences by age in vocalization duration (albeit less than the hearing age matched group). So, the children with CIs vocalize just as frequently as TH groups and the cross-sectional comparison suggests developmental progression. Nevertheless, given the differences in vocalization duration between the children with CIs and chronological matches, and the significantly weaker effect of age among the children with CIs, it appears that the children with CIs follow a different vocal pattern than either TH group. It could be that children with CIs produce shorter vocalizations but supplement them with other modalities (e.g., gestures). It could be that they follow a non-linear developmental trend, and it seems clear that the developmental trajectory would vary by the child’s implantation date. Nevertheless, note that this conclusion differs from a number of studies looking at short-term changes in vocal productivity following cochlear implant activation which have found that children produce developmentally-appropriate (for their hearing age) amounts of canonical and reduplicated babble months post-activation (Fagan, 2014; Schauwers et al., 2008). The duration of a child’s vocalizations, however, reflects a number of different components in speech development: how long can a child sustain phonation, how many sequential syllables can the child produce, how many phonemes is the child producing, etc. and as such is a different metric of speech development than babbling landmarks. It is also possible that the duration of a child’s vocalizations could stay the same while the internal structure of each vocalization becomes more phonologically complex. The vocal development findings from Fagan, Schauwers, and other colleagues also stemmed from shorter observations (ranging 15–80 mins/child), often collected in the lab. Going forward we plan to evaluate vocal maturity in this maximally naturalistic dataset that we have collected using a combination of vocal maturity algorithms and hand-coding which should allow us to compare our results more directly with previous research.

At-home language interventions among children with TH often target caregiver input (caregiver-child conversational turns: Ferjan Ramírez et al. (2020); Romeo et al. (2021); caregiver words: Suskind et al. (2016); or both: Suskind et al. (2013)). But child vocal productivity partially *drives* caregiver input (Warlaumont et al., 2014). Indeed, a number of interventions for groups with different developmental profiles, including less verbal profiles such as children with autism spectrum disorder diagnoses or classic galactosemia, instead target the child’s own vocal productions (Peter et al., 2021). Results here suggest that preschoolers with CIs are not necessarily lacking speech input – there were no differences in overall amounts of input by group (though quantity of input vs. intake could differ). Instead, the children may require increased opportunity for their own vocal practice in order to progress developmentally and hit speech production landmarks. It also seems likely that children with CIs may require more input relative to children with TH, or at least that the input be more audible (e.g., free from background noise).

### Caregiver input and child vocal output

Infants and children speak more when spoken to. Yet children with CIs may be less likely to notice when a caregiver is speaking to them, distinguish caregiver speech input from distractors, and parse individual words because CIs compromise sound localization, speaker identity, and prosodic cues (Chatterjee & Peng, 2008; Todd et al., 2016). This was the result that we found – the daily speech-language environment was *less* predictive of vocal productivity for children with CIs than either TH group.

Children with CIs consistently underperform their peers with TH on almost every measure of speech, language, and literacy (Mayer & Trezek, 2018; Nittrouer & Caldwell-Tarr, 2016). This speech-language gap persists into adolescence and even adulthood (Breland et al., 2022). And while some children with CIs develop stronger speech-language skills than others – nearly on par with their TH peers (Niparko et al., 2010) – even 4–6 years post-implantation, more than 50% of child CI 519 recipients perform 1–2 standard deviations below peers on many standardized measures of speech and language (Eskridge et al., 2021; Karltorp et al., 2020). Since the richness of the home language environment is a strong predictor of future speech, language, and literacy outcomes for a variety of populations of children with TH (Swanson et al., 2019; Weisleder & Fernald, 2013), and child CI recipients are at risk of speech and language delays, providing caregiver counseling to optimize the home language environments of children with CIs could be a promising method to close the spoken language gap for these children. These interventions could take the form of clinicians encouraging parents to reflect on which times of day they interact the most with their child, or to note how their child responds more readily when there isn’t a large amount of background noise interference. Many unknowns remain before such interventions could reliably be implemented, but the work we present here is an important step towards making at-home interventions a reality. Demonstrating how the home environment systematically differs by hearing status affects how clinical interventions to *shape* the home linguistic environment should be implemented. For example, our results suggest that the daily speech environment is less predictive of vocal productivity among children with CIs than children with TH. So we might not expect interventions targeting increased caregiver-child vocal interaction to elicit the same vocal productivity benefit for preschoolers with CIs as those with TH; instead, we should be evaluating intervention success based on the unique input-outcome relationships for children with CIs, such as those documented here. Finally, it is possible that the child’s speech-language education program (e.g., Total Communication, Auditory-Verbal Therapy) could also interact with these caregiver-child vocal interactions, as well as with any potential behavioral interventions. Many auditory-verbal therapy programs, for example, emphasize an active role for caregiver-initiated spoken language stimulation. Thus, an interesting avenue for future research could be to see how the impact of caregiver language interventions differ by the child’s education program.

### Limitations

Sampling biases are a concern for developmental research in general (Singh et al., 2022), but may be especially prevalent for methodologies such as at-home recordings since some families are unwilling to record in their homes. Marginalized groups with a history of being tracked may, understandably, be especially wary. As such, samples in developmental science that already skew white and middle to upper class may be especially biased for at-home methods. Thus, we stress that current findings about e.g., the strength of the relationship between adult input and child vocal production may not generalize to all children, even within North America.

Another limitation of this study is that recordings were only taken on one day of the child’s life. Although our own work and others’ suggests that differences across days WITHIN households are less than differences BETWEEN households (de Barbaro & Fausey, 2022), future work could more comprehensively address this concern by ensuring that all families record over two or three days, as some others have done (Romeo et al., 2018). Another way to ensure sampling consistency, but instead between families, would be to request that families record, for example, on both one weekend and week day, or for an entire weekend. Observing each child for 16 hrs. already lends insight that more limited, in-lab observations cannot; yet collecting even denser samples than those we present here – those that span longer time periods – could ensure that the observations made on a single day in the child’s life are not biased by exceptional events. It is likewise important to note that we did not have access to reports of children’s daily device use (from data logs). As such, we may have been observing children in their homes during times when the devices were not being worn (although we encouraged caregivers to make sure that the devices were worn during all LENA observation). Ideally, future research on this topic would be able to report on both LENA measures and hours of device use.

## Conclusion

This work evaluated the daily speech environments of preschoolers with cochlear implants in comparison to two groups of their peers with typical hearing. Using incredibly dense sampling of children’s everyday environments in their homes, we assessed how a battery of everyday speech-language experiences – caregiver speech input, child vocal production, and caregiver-child conversational turns – differed by hearing status. Take-aways are that (1) the speech-language environment reflects development less closely for children with implants than typical hearing and (2) there were minimal differences by hearing status in caregiver-child interaction, even after implementing the measure in multiple ways. The unique auditory experiences of preschoolers who receive cochlear implants, the time they spend without auditory access pre-implantation and the degraded device signal they learn from post-implantation, do shape their everyday speech and language environments.

## Supplementary Material

supplementary materials I

supplementary materials II

## Figures and Tables

**Table 1.** Demographic and audiological information. Mean (SD), range.

	Chrono. age matches	Cochlear implant	Hearing age matches
Chrono Age (mos)	46.28 (10.8), 32–66	47.72 (9.84), 31–65	35 (12.71), 17–52
Gender (F, M)	9,9	9,9	9,7
Mat. Ed.	6.22 (1), 3–7	6.11 (1.02), 3–7	6.25 (1), 3–7
Hearing Age (mos)	NA	31.28 (14.3), 8–54*	NA
Activation Age (mos)	NA	16.44 (9.7), 7–45	NA
Num. of siblings	1.61 (0.98), 1–5	1.39 (0.85), 0–3	1.12 (0.96), 0–3
Num. of household members	4.56 (1.04), 3–8	4.33 (0.97), 2–6	4.06 (1.06), 2–6
Ethnicity (N)			
Hispanic	2	0	2
Race (N)			
Asian	0	1	2
Black	2	0	0
White	14	14	13
American Indian	1	0	0
Asian & white	0	0	1
Black & white	0	1	0
More than 1 race (unspecified)	1	0	0

* Includes the 2 children with cochlear implants who had hearing ages < 12 mos and were thus not included in those matched by hearing age to children with typical hearing.

† Ethnicity information was unavailable for one child with implants and race information unavailable for two children with implants.

**Table 2.** Measures of the naturalistic speech environment, by hearing group. Mean(SD), range

	Chrono. age matches	Cochlear implant	Hearing age matches
Recording duration (hrs)	15.82(0.75), 12.83–16	16(0), 16–16	16(0), 16–16
Input			
Adult speech/hr (words)	1081.49(481.29), 285.63–2250.39	1217.23(508.87), 411.36–2127.7	1105.54(433.01), 170.88–1630.86
Adult speech/hr (s)*	258.36(118.69), 72.79–533.67	288.52(121.52), 95.31–499.53	264.55(102.57), 43.67–386.22
Adult word consistency	0.52(0.13), 0.31–0.78	0.58(0.11), 0.25–0.7	0.51(0.12), 0.19–0.66
Output			
Child voc. quantity	308.03(142.81), 90.12–575.81	271.75(69.23), 48.75–381.62	254.5(108.83), 42.5–424
Voc. duration (ms)	1004.46(662.3), 80–10940	937.93(569.76), 80–13270	966.59(627.6), 80–19730
Child voc. consistency	0.55(0.15), 0.34–0.84	0.58(0.13), 0.17–0.72	0.49(0.14), 0.22–0.69
Interaction			
Convo. turn quantity	61.71(32.78), 20.69–150.94	68.17(26.47), 8.5–116.75	65.13(25.47), 11.12–92.62
Convo. turn consistency	0.58(0.14), 0.38–0.84	0.64(0.13), 0.22–0.77	0.56(0.12),0.36–0.74

* Descriptive statistics are reported for seconds of speech/hour, but modeling was conducted on minutes of speech/hour.

**Table 3.** Relationship between experience (age, in mos) and measures of the naturalistic speech environment, by hearing group.

	Chrono. age matches	CI chrono. age	CI hearing age	Hearing age matches
Adult words	*β*=20.7+ r=0.46	*β* = 3.81 r = 0.07	*β* = 0.77 r = 0.02	*β* = 16.49+ r = 0.48
Adult speech (s)	*β* = 5.27* r = 0.48	*β* = 1.12 r = 0.09	*β* = 0.31 r = 0.03	*β* = 3.78+ r = 0.47
Child voc. quantity	*β* = 3.98 r = 0.34	*β* = 2.71 r = 0.33	*β* = 1.06 r = 0.17	*β* = 8.03*** r = 0.89
Child voc. duration (s)	*β* = .59 r = 0.02	*β* = 3.16* r = 0.05	*β* = 1.84 r = 0.03	*β* = 6.59** r = 0.11
Convo. turn	*β* = 0.83 r = 0.29	*β* = 0.01 r = 0	*β* = −0.33 r = −0.16	*β* = 2.18*** r = 0.93

β=model coefficient from linear regression, p-value from linear model parameter

*** p <.001

** p <.01

* p <.05)

+ p <.1, r=Pearson correlation coefficient. No p-value annotation indicates p >. 1

## Data Availability

All data and code used to generate the analyses in this paper are publicly available at: https://github.com/megseekosh/everyday_CI.

## Appendix

**Appendix Table A1:** Audiological information from children with cochlear implants

Participant	Chronological age	Age at hearing loss (mos)	Age at activation	Hearing age (since activation)	Etiology	Device configuration	Activation
300ECV1	58	0	13	45	genetic	bilateral	simultaneous
301ECV1	53	0	45	8	unknown	bilateral	R-L
302ECV1	37	0	13	24	unknown	bilateral	R-L
303ECV1	65	6	13	52	unknown	bilateral	simultaneous
304ECV1	48	0	12	36	genetic	bilateral	R-L
307ECV1	44	0	15	29	genetic	bilateral	R-L
308ECV1	39	0	13	26	genetic	bilateral	simultaneous
311ECV1	62	9	13	49	unknown	bilateral	L-R
312ECV1	44	0	24	20	genetic	unilateral	R
314ECV1	38	10	17	21	unknown	bilateral	R-L
801ECV1	39	1.5	15	24	unknown	bilateral	simultaneous
804ECV1	56	0	7	49	genetic	bilateral	simultaneous
806ECV1	45	14	34	11	genetic	unilateral	L
807ECV1	51	10	22	29	Mondini malformation	bimodal	n/a
309ECV1	61	0.5	7	54	genetic	bilateral	simultaneous
306ECV1	49	0	8	41	unknown	bilateral	R-L
605LTP1	31	0	16	15	unknown	bimodal	n/a
608LTP1	39	0.5	9	30	Connexin 26	bilateral	simultaneous

Information about the specific dB of hearing loss was not available.
