# Supplementary material for: The everyday speech environments of preschoolers with and without cochlear implants
Source: J Child Lang. Author manuscript; Available in PMC 2025 Mar 1. (PMC11327381; doi:10.1017/S0305000924000023)
Supplement: supplementary materials I [file NIHMS1990848-supplement-supplementary_materials_I.pdf]

Supplementary Materials I for “The everyday speech environments of preschoolers with and without cochlear implants”

Family composition details by hearing group

Table 1

Detailed household composition information

	Chrono. age matches	Cochlear implant	Hearing age matches
**Num. of siblings**	1.61 (0.98), 1-5	1.39 (0.85), 0-3	1.12 (0.96), 0-3
0	0	2	4
1	10	9	8
2	7	5	2
3	0	2	2
4	0	0	0
5	1	0	0
**Num. of household members**	4.56 (1.04), 3-8	4.33 (0.97), 2-6	4.06 (1.06), 2-6
**Birth order**			
Youngest	5	6	8
Middle	3	3	0
Oldest	4	6	2
Only child	1	2	5
Twin	0	0	1
Not available	5	1	0
