# Supplementary material for: The everyday speech environments of preschoolers with and without cochlear implants
Source: J Child Lang. Author manuscript; Available in PMC 2025 Mar 1. (PMC11327381; doi:10.1017/S0305000924000023)
Supplement: supplementary materials II [file NIHMS1990848-supplement-supplementary_materials_II.pdf]

Supplementary Materials II for "The everyday speech environments of preschoolers with and without cochlear implants"

Replicating results after removing child activated at 45 mos

Input

We quantified the children's speech-language input by computing the average number of minutes/hour that contained speech from an adult female or male near the child. We additionally computed the average number of words/hour spoken by an adult near the child.

For all examples of repeated measures, we fit linear mixed effects models with random intercepts by child and a fixed effect of **Hearing Group**. Models of hourly measures (words, minutes) additionally included random intercepts by hour of recording. There were no reliable differences by **Hearing Group** for measures of input quantity (hourly words, hourly minutes of speech; log-likelihood tests all p >.05); thus, all hearing groups received similar amounts of input in the environment (words and minutes).

We evaluated the consistency of speech input by hearing group by computing the percentage of minutes in each recording containing $\geq$ 1 word from an adult. There were no differences in speech input consistency by **Hearing Group** (p >.05). However, speech input became more *consistent* with **Child Age** (age coded continuously, in months) across the entire sample, independent of hearing status (model fit: β=0.004, t=3.30, p=.002). This result indicates that speech is more continuously present throughout the day in older children. Note that this measure of consistency is independent of speech quantity, or the overall *amount* of speech input (words or minutes), which we explore below. Speech input is more consistent—more evenly spread out and less clustered into bursts over the course of the day—in older children across the sample.

Finally, we evaluated differences by hearing group in a cross-sectional analysis of speech input by age. For this analysis, we modeled the effect of **Child Age** (in mos) upon hourly adult word token count and minutes of adult speech/hour in the children's environments. Hourly word counts and minutes of speech/hour increased with child age in both groups of children with TH, but not by hearing or chronological age among the children with CIs: for every month of development, chronological age matches (spanning 32-66 mos) received approximately 21 additional words/hour and 5 additional seconds of speech/hour while hearing age matches (17-52 mos) received an

additional 16 words/hour and 4 seconds of speech/hour. Again, no such cross-sectional effect by age was seen for the children with CIs.

Output

To assess each child's speech output (production), we computed the average number of vocalizations from the target child spoken/hour. We additionally analyzed the impact of hearing group upon the children's vocalization intensity and duration.

Once again for the repeated measures (vocalization duration), we fit linear mixed effects models with random intercepts by child and a fixed effect of **Hearing Group**. Models of the hourly vocalizations additionally included random intercepts by hour of recording. There was no effect of **Hearing Group** on the number of vocalizations/hour (p >.05); so, hearing status did not dictate the amount of the children's speech. However, there was an effect of hearing status in the model predicting vocalization duration (comparison of models with and without **Hearing Group**: χ^2=6.72, df=2, p=.03): the chronological age matches produced significantly longer vocalizations than both the children with CIs (β=57.62) and hearing age matches (β=78.72).

We additionally measured the consistency of children's speech output which we quantified as the percentage of minutes in each recording containing at least one vocalization from the target child; there was no effect of hearing experience upon children's vocalization output consistency.

Finally, we measured the cross-sectional differences by age in vocalization quantity and duration: there was a significant, positive effect of **Child Age** (mos) on vocalization duration among the children with CIs by chronological age, and for the hearing age matches. With each additional month, the duration of the children with CIs' vocalizations increased by approximately 3.31ms, a shallower slope than for the hearing age-matched children with TH (6.59ms/month).

Interaction

We next evaluated the impact of hearing group upon caregiver-target child interactions. There was no effect of **Hearing Group** upon the quantity or consistency of turns (both $p > .05$).

The cross-sectional analysis by age showed a positive relationship between age and conversational turn quantity only for the hearing age matches (e.g. for the youngest children).

Predicting vocal productivity from input measures

For the final analysis, we examined how measures of the speech environment predicted children's speech productivity and how this varied by hearing group. The measure of the speech environment that we examined was the **Average number of conversational turns/hr**. The measure of speech productivity that we used was the average number of target child vocalizations/hour in each recording.

It is expected that children who hear more speech, and engage in more linguistic interactions with caregivers, should vocalize more. We aimed to evaluate how this relationship between conversational turns and child vocal productivity might differ by hearing status. The interaction of **Average number of conversational turns/hour** and **Hearing Group** did indeed improve upon a model without the interaction (model fit comparison: χ^2=3.65, df=2, p= .03), suggesting differences in the strength of this relationship by hearing status. Modeling demonstrated that for every additional conversational turn per hour that children with CIs engaged in, they produced approximately two additional vocalizations per hour (β=2.11, t=4.48, p<.001). However, this relationship between turns and child vocal productivity was significantly steeper for both groups of children with TH who produced approximately 3 or 4 additional vocalizations per hour for every hourly conversational turn that they engaged in (chronological matches: β=1.55, t=2.54, p=.01, or a slope of 3.66; hearing age matches: β=1.41, t=2.21, p=.03, or a slope of 3.52).
